# Myelin-phagocytosing macrophages modulate autoreactive T cell proliferation

**DOI:** 10.1186/1742-2094-8-85

**Published:** 2011-07-25

**Authors:** Jeroen FJ Bogie, Piet Stinissen, Niels Hellings, Jerome JA Hendriks

**Affiliations:** 1 Hasselt University/Transnational University Limburg, School of Life Sciences, Biomedical Research Institute, Diepenbeek, Belgium

## Abstract

**Introduction:**

Multiple sclerosis (MS) is a chronic, inflammatory, demyelinating disease of the central nervous system (CNS) in which macrophages play a central role. Initially, macrophages where thought to be merely detrimental in MS, however, recent evidence suggests that their functional phenotype is altered following myelin phagocytosis. Macrophages that have phagocytosed myelin may be less inflammatory and may exert beneficial effects. The presence of myelin-containing macrophages in CNS-draining lymph nodes and perivascular spaces of MS patients suggests that these cells are ideally positioned to exert an immune regulatory role. Therefore we evaluated in this study the effect of myelin-phagocytosing macrophages on lymphocyte reactivity.

**Methods:**

Thioglycolate-elicited rat peritoneal macrophages were loaded with myelin and cocultured with myelin-basic protein (MBP) or ovalbumin (OVA) reactive lymphocytes. Lymphocyte proliferation was determined by CFSE-labeling. The role of nitric oxide in regulating lymphocyte proliferation was assessed by addition of an inhibitor of inducible nitric oxide synthase to the coculture. *In vivo *immune regulation was investigated by treating MBP- and OVA-immunized animals subcutaneously with myelin. Cognate antigen specific lymphocyte proliferation and nitric oxide production were determined 9d post-immunization.

**Results:**

In this study we demonstrate that myelin-phagocytosing macrophages inhibit TCR-triggered lymphocyte proliferation in an antigen-independent manner. The observed immune suppression is mediated by an increase in NO production by myelin-phagocytosing macrophages upon contact with lymphocytes. Additionally, myelin delivery to primarily CD169^+ ^macrophages in popliteal lymph nodes of OVA-immunized animals results in a reduced cognate antigen specific proliferation. In contrast to OVA-immunized animals, lymphocytes from MBP-immunized animals displayed an increased proliferation after stimulation with their cognate antigen, indicating that myelin-phagocytosing macrophages have dual effects depending on the specificity of surrounding lymphocytes.

**Conclusions:**

Collectively our data show that myelin phagocytosis leads to an altered macrophage function that inhibits lymphocyte proliferation. Additionally, results from this study indicate that myelin-phagocytosing macrophages fulfill a dual role *in vivo*. On one hand they aggravate autoimmunity by activating myelin-reactive lymphocytes and on the other hand they suppress lymphocyte reactivity by producing NO.

## Introduction

Multiple Sclerosis (MS) is characterized as a chronic, inflammatory, neurodegenerative disease of the central nervous system (CNS). It is regarded to be an autoimmune disease as activated autoimmune lymphocytes are pivotal in orchestrating the immunopathological processes involved in myelin sheath damage [1-4].

Pathologically, MS is characterized by CNS infiltration of activated myelin-reactive lymphocytes and macrophages, resulting in an inflammatory microenvironment. Microglia and macrophages typically accumulate in the perivascular spaces and the brain parenchyma near terminal ovoids of transected axons [5]. They are thought to be the primary effector cells in MS and its animal model, experimental allergic encephalomyelitis (EAE) [6-8]. Effector mechanisms of activated macrophages and microglia include the internalization of myelin, and the secretion of inflammatory and toxic mediators which negatively influence axonal and myelin integrity [9-22].

In contrast to their apparent detrimental role in MS, increasing evidence suggests an additional neuroprotective role for macrophages. Although two seemingly mutually exclusive processes, various studies have reported such a dual role of monocytes and macrophages in both injury and repair [23,24]. In neurodegenerative models, remyelination is for instance often correlated with large numbers of macrophages and microglia in an inflammatory microenvironment [25-27]. Furthermore, as contact with CNS myelin debris inhibits oligodendrocyte progenitor maturation *in vitro*, and as macrophages have been described to actively phagocytose myelin debris, local clearance of myelin debris in the centre or vicinity of lesions is suggested to be a necessary prerequisite for axonal remyelination following demyelination [28]. This hypothesis is supported by the fact that monocyte depletion and a consequent inability to clear the microenvironment of myelin debris, causes an impairment of oligodendrocyte progenitor differentiation *in vivo *[29,30]. Finally, recent evidence indicates that monocyte-derived macrophages, peritoneal macrophages, microglia and dendritic cells (DCs) obtain anti-inflammatory characteristics following internalization of myelin [12-14,31]. These studies clearly demonstrate that macrophages, besides their apparent role in neurodegeneration, may exert a neuroprotective influence on MS pathogenesis by clearance of myelin debris and by altering their phenotype following myelin internalization.

Perivascular macrophages, infiltrated macrophages and microglia are ideally positioned to influence infiltrating and infiltrated myelin-reactive lymphocytes. Indeed, CNS reactivation of autoreactive lymphocytes by local antigen presenting cells displaying myelin antigens is thought to initiate and maintain the inflammatory cascade observed in the brain of MS patients [3,4,32]. The presence of brain antigen-containing phagocytes in secondary lymph nodes in MS and EAE further emphasizes a possible crucial role of these cells in modulating the immune response during MS and EAE pathogenesis [33-35]. Phenotypical analysis of these macrophages further revealed that in contrast to neuronal antigen containing phagocytes, the majority of myelin-containing APCs express anti-inflammatory mediators. How brain antigens gain excess to CNS draining secondary lymph nodes, either chemotactically in the context of phagocytes or as soluble products, remains to be clarified [12,36,37].

In this study we investigated the capacity of myelin-phagocytosing macrophages (mye-macrophages) to influence lymphocyte proliferation. We show that mye-macrophages inhibit TCR-triggered lymphocyte proliferation in an antigen-independent manner. This process is mediated by an enhanced nitric oxide (NO) production. Furthermore, we demonstrate that myelin delivery to popliteal lymph nodes of OVA-immunized animals and uptake by primarily CD169^+ ^macrophages reduces cognate antigen specific proliferation following restimulation *ex vivo*. The elevated production of NO detected in these lymph node cultures indicates that NO may also mediate the immune suppressive effects *in vivo*. In contrast, myelin delivery to popliteal lymph nodes did increase lymphocyte reactivity in MBP-immunized animals. Thus, mye-macrophages may play a suppressive role in CNS-draining lymph nodes during MS pathogenesis, depending on the nature of surrounding lymphocytes. Collectively our data provide evidence that myelin phagocytosis leads to an altered macrophage function that modulates lymphocyte responses.

## Methods

### Animals

Female Lewis rats, 6-8 weeks of age, were purchased from Harlan Netherlands B.V. (Horst, The Netherlands). Animals were housed in the animal facility of the Biomedical Research Institute of Hasselt University. Experiments were conducted in accordance with institutional guidelines and approved by the local Ethical Committee for Animal Experiments of Hasselt University.

### Isolation of peritoneal rat macrophages

Three days prior to macrophage isolation, rats were injected intraperitoneally with 3 ml 3% thioglycolate (Sigma-Aldrich, Bornem, Belgium). Resident peritoneal macrophages were obtained by peritoneal lavage using 10 ml of ice-cold PBS (Lonza, Vervier, Belgium) supplemented with 5 mM ethylenediamine tetraacetic acid (EDTA; VWR, Leuven, Belgium). Peritoneal exudate cells (PECs) were cultured for 2 hours in RPMI 1640 medium. After 2 hours incubation at 37°C with 5% CO_2_, non-adherent cells were washed away. Remaining cells were >95% macrophages [38].

### Myelin phagocytosis

Myelin was purified from rat brain tissue by means of density-gradient centrifugation, as described previously [39]. Myelin protein concentration was determined by using the BCA protein assay kit (Thermo Fisher Scientific, Erembodegem, Belgium). LPS content was determined using the Chromogenic Limulus Amebocyte Lysate assay kit (Genscript Incorperation, Aachen, Germany). Isolated myelin contained a neglectable amount of endotoxin (1.8 × 10^-3 ^pg/μg myelin).

Isolated myelin was fluorescently labelled, according to the method of Van der Laan et al. [11]. In short, 10 mg/ml myelin was incubated with 12.5 μg/ml 1,1"-diotadecyl-3,3,3',3',-tetramethylindocarbocyanide perchlorate (DiI; Sigma-Aldrich) for 30 min at 37°C. Next, a myelin phagocytosis assay was performed as previously described [39].

### Immunization and *in vivo *myelin treatment

Lewis rats were injected subcutaneously with a 0.1 ml suspension containing 250 μg/ml guinea pig myelin basic protein (MBP) or ovalbumin (OVA), 2.5 mg/ml H37RA heat-killed mycobacterium tuberculosis (Difco, Detroit, USA) and 60 μl Complete Freunds adjuvant (Sigma-Alldrich) in both hind paws. Subsequently animals were injected subcutaneously with PBS, 2.6 × 10^6 ^latex beads (0.8 μm mean particle size, Sigma-Alldrich), 75 μg/animal of isolated myelin or OVA (d-4, 0, 4 and 8 pre- and post-immunization). MBP-immunized rats were weighted and scored daily according to the following neurological scale: 0 = no neurological abnormalities, 0.5 = partial loss of tail tonus, 1 = complete loss of tail tonus, 2 = hind limb paresis, 3 = hind limb paralysis, 4 = moribund, 5 = death.

### Generation of antigen-specific lymphocytes

MBP and OVA-specific lymphocytes were obtained 9 days post-immunization by bilateral isolation of the inguinal and popliteal lymph nodes. Single-cell suspensions of harvested lymph nodes were obtained by grinding with a syringe plunger against a 70 μm cell strainer (Bellco Glass Inc., Vineland, USA). To enrich for antigen specific lymphocytes, lymph node cells were restimulated, as described previously [40]. Briefly, lymph node cells were resuspended in stimulation medium: RPMI 1640 medium (Invitrogen, Merelbeke, Belgium) supplemented with 50 U/ml penicillin (Invitrogen), 50 U/ml streptomycin (Invitrogen), 20 μM 2-mercapto-ethanol (Sigma-Alldrich), 1% sodium pyruvate (Invitrogen), 1% MEM non-essential amino acids (Invitrogen), 2% deactivated autologous serum and 33 μg/ml MBP. After 2 days, cells were washed and resuspended in RPMI 1640 medium supplemented with 50 U/ml penicillin, 50 U/ml streptomycin, 20 μM 2-mercapto-ethanol, 10% fetal calf serum (FCS, Hyclone, Erembodegem, Belgium) and 6,5% supernatants of Concanavalin (ConA, Sigma-Alldrich) stimulated spleen cells. Following 2 days, cells were washed and resuspended in RPMI 1640 medium supplemented with 50 U/ml penicillin, 50 U/ml streptomycin, 20 μM 2-mercapto-ethanol and 10% fetal calf serum for 3 days.

### CFSE-labeling of lymphocytes

A carboxyfluorescein diacetatesuccinimidyl ester (CFSE) stock (10 mM in DMSO, Invitrogen, Merelbeke, Belgium) was diluted in PBS (Biowhittaker™). Antigen-specific lymphocytes were resuspended in PBS supplemented with 0.05% BSA and 4 μM CFSE (20 × 10^6 ^cells/ml) for 7 min at 37°C with 5% CO_2_. Cells were washed and diluted in 0.5 ml culture medium for 30 min at 37°C with 5% CO_2 _to stabilize the CFSE-labeling. In parallel, to determine macrophage viability following a coculture with lymphocytes, macrophages were labeled with CFSE to distinguish them from unlabeled lymphocytes.

### Coculture of macrophages with lymphocytes

Prior to coculture with CFSE labeled lymphocytes, isolated macrophages were seeded in flat-bottem 96-well plates (15 × 10^3 ^cells/well) in RPMI 1640 medium supplemented with 50 U/ml, 50 U/ml streptomycin and 10% FCS, and treated with 100 μg/ml of isolated myelin for three hours. Excess myelin was removed by washing twice with RPMI 1640 medium at 37°C. Subsequently, stimulation medium containing irradiated thymocytes (15 × 10^4^, 3000 rad), CFSE-labeled MBP- or OVA-specific lymphocytes (15 × 10^4^) and respectively 10 μg/ml MBP or 10 μg/ml OVA were added. Untreated macrophages were used as a control. To evaluate the involvement of respectively NO, arginase, indoleamine 2,3-dioxygenase (IDO) the phagocytosis process itself, direct cell-cell contact and IFNγ, 1.5 mM N^G^-Monomethyl-L-arginine (L-NMMA; VWR), 0.5 mM N^G^-Hydroxy-L-arginine (NOHA; VWR), 0.2 mM 1-Methyl-L-tryptophan (1-MT; Sigma Alldrich), latex beads (1:100), 100 μg/ml zymosan A (Sigma-Alldrich), transwell inserts (0.4 μm pore size, Sigma-Aldrich) or 10 μg/ml anti-rat IFNγ (Preprotech, London, UK) were tested in the coculture model.

Flow cytometry was used to assess proliferation and cell death of lymphocytes and macrophages after a 4 day coculture. Here, cells were stained with PE-conjugated mouse-anti-rat CD3 (Immunosource, Erembodegem, Belgium) or CD11b (AbD Serotec, Düsseldorf, Germany) and 7 aminoactinomycin D (7AAD, BD Biosciences).

### [^3^H]Thymidine incorporation

Isolated lymph node cells (20 × 10^4^) were cultured with MBP (10 μg/ml), OVA (10 μg/ml) or myelin-oligodendrocyte glycoprotein (MOG, 20 μg/ml). Additionally, 100 μg/ml of isolated myelin was added in some experiments. Following 48 hr, 1 μCi [^3^H]thymidine (Amersham, Buckinghamshire, UK) was added to the culture. Next, cells were harvested with an automatic cell harvester (Pharmacia, Uppsala, Sweden) and uptake of radioactivity was measured in a β-plate liquid scintillation counter (Wallac, Turku, Finland).

### Nitrite formation

Coculture supernatants were collected and release of NO was determined using the griess reagent system (Promega, Leuven, Belgium), following the manufacturer's instructions. Absorbance was determined by using a microplate reader at 550 nm (Biorad Benchmark).

### Histology and immunohistochemistry

Snap-frozen brain and spinal cord material was cut in respectively the coronal and sagittal plane with a Leica CM1900UV cryostat (Leica Microsystems, Wetzlar, Germany) to obtain 10 μm sections. The extent of demyelination and infiltration was determined by staining with Luxol Fast Blue (LFB; Gurr BDH, Poole, England). Briefly, aceton-fixed slides were incubated with LFB for 16 hr at 56°C, destained with 0.05% lithium carbonate, and counterstained with cresyl violet (VWR). Analysis was carried out using a Nikon eclipse 80i microscope and NIS Elements BR 3.10 software (Nikon, Tokyo, Japan).

DiI-labeled myelin migration to popliteal and inguinal lymph nodes was determined by immunohistochemistry. Popliteal and inguinal lymph nodes were snap-frozen directly following isolation and cut into 10 μm sections. Following fixation in respectively aceton for 10 min, sections and cells were blocked using 10% goat serum (Millipore, Brussels, Belgium) in PBS. Subsequently, sections and cells were stained with mouse-anti-rat CD169 (1/250 in PBS; Abd Serotec), a marker for macrophages in lymph nodes. As a secondary antibody Alexa fluor 488 F(ab')_2 _fragment of goat-anti mouse was used (1/500 in PBS; Invitrogen). Control staining was performed by omitting the primary antibody. Nuclear staining was performed using 4,6'-diamidino-2-phenylindole (DAPI; Invitrogen) for 10 min. Autofluorescence was minimalized by using 0.1% Sudan Black in 70% ethanol.

### Statistical analysis

Data were statistically analyzed using GraphPad Prism for windows (version 4.03) and are reported as mean ± SEM. D'Agostino and Pearson omnibus normality test was used to test normal distribution. An analysis of variances (ANOVA) or two-tailed unpaired student T-test (with Welch's correction if necessary) was used for normally distributed data sets. The Kruskal-Wallis or Mann-Whitney analysis was used for data sets which did not pass normality. *P < 0,05, **P < 0,01 and ***P < 0,001.

## Results

### Myelin-laden macrophages inhibit lymphocyte proliferation

**Figure 1:**
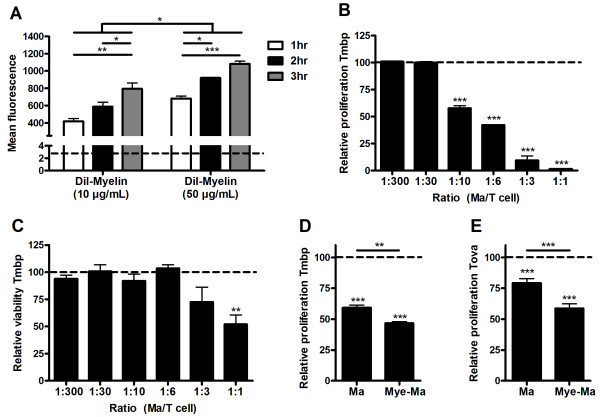
**Myelin-phagocytosing macrophages inhibit TCR-triggered lymphocyte proliferation in an antigen-independent manner**. (a) Mean DiI-flourescence following treatment of macrophages with divergent concentrations of myelin (10 and 50 μg/ml) for several periods of time (1 hr; white bars, 2 hr; black bars or 3 hr; grey bars). The dotted line represents untreated macrophages. Data represent the mean of two independent experiments. (b, c) Lymphocyte proliferation and viability following a 4d coculture with irradiated thymocytes, purified MBP and different concentrations of macrophages. The relative proliferation and viability is defined as the percentage of proliferating or viable cells in experimental cultures divided by values in control lymphocyte cultures without macrophages (dotted line). Data represent the mean of three independent experiments. (d, e) Proliferation of MBP or OVA-reactive lymphocytes following a 4d coculture with irradiated thymocytes, macrophages or mye-macrophages (macrophage/lymphocyte ratio 1/10) and respectively purified MBP or OVA. Data represent the mean of six (d) and four (e) independent experiments. Dotted line represents the proliferation of control lymphocyte cultures without macrophages. Ma: Macrophages.

Initially we assessed the capacity of peritoneal macrophages to internalize myelin. By culturing macrophages with different concentrations of DiI-labeled myelin for divergent periods of time, it was demonstrated that myelin is internalized in a time- and dose-dependent manner (Figure 1a). Next, the most optimal macrophage/lymphocyte coculture ratio was determined by using untreated macrophages. At high macrophage/lymphocyte ratio's, lymphocytes demonstrated a reduced viability and proliferation (Figure 1b and 1c). The decline of lymphocyte viability was absent at low macrophage/lymphocyte ratio's (<1/10). To observe differences in lymphocyte proliferation following coculture with mye-macrophages, subsequent experiments were conducted using a macrophage/lymphocyte ratio of 1/10.

To study whether mye-macrophages affect antigen-specific lymphocyte proliferation in a different manner compared to untreated macrophages, macrophages or mye-macrophages were cocultured for 4d with MBP- or OVA-reactive lymphocytes, irradiated thymocytes and purified MBP or OVA. Here it was demonstrated that mye-macrophages inhibit TCR-triggered lymphocyte proliferation more pronounced than untreated macrophages (Figure 1d and 1e). This process was independent of antigen-specificity, since both MBP and OVA-reactive lymphocytes showed the same reduction in proliferation. Proliferation differences were not due to an altered viability of lymphocytes or mye-macrophages (data not shown).

### Inhibition of lymphocyte proliferation is independent of the phagocytosis process or myelin-antigen presentation

**Figure 2:**
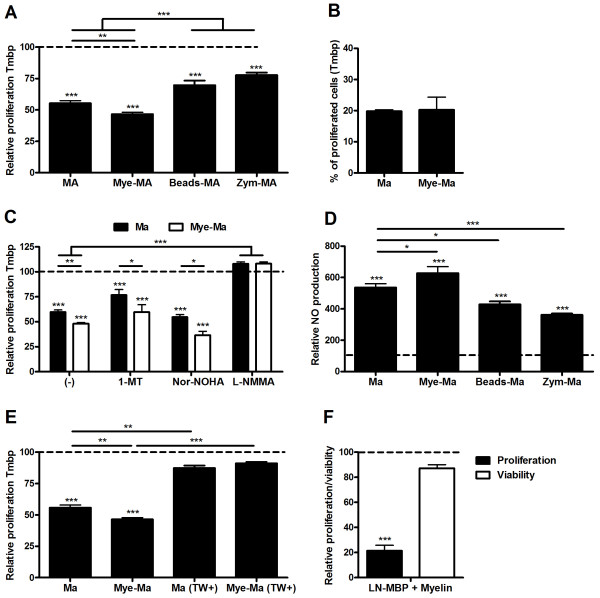
**Nitric oxide secreted by myelin phagocytosing macrophages inhibits lymphocyte proliferation *in vitro***. (a) Comparison of lymphocyte proliferation following coculture with untreated, myelin-, zymosan- (Zym) or latex bead-treated macrophages. The relative proliferation is defined as the percentage of proliferating cells in experimental cultures divided by values in control lymphocyte cultures without macrophages (dotted line). Data represent the mean of two independent experiments. (b) To determine the capacity of mye-macrophages to present myelin antigens, MBP-reactive lymphocytes were cocultured with macrophages or mye-macrophages (ratio 1/10) in the absence of thymocytes and MBP. Data represent the mean of two independent experiments. (c) The role of respectively IDO, arginase and NO in the observed inhibition of proliferation, macrophage coculture (black bars) and mye-macrophage coculture (white bars), was determined by addition of an IDO inhibitor (1-MT), an arginase inhibitor (Nor-NOHA) and an iNOS inhibitor (L-NMMA). Dotted line represents the proliferation of control lymphocyte cultures without macrophages. Data represent four independent experiments. (d) Relative NO concentration in supernatants following a 4d coculture with MBP-reactive lymphocytes. Dotted line represents the NO production of control lymphocyte cultures without macrophages. Data represent the mean of 4 independent experiments. (e) Comparison of lymphocyte proliferation following coculture with untreated and myelin-treated macrophages using transwell inserts. Dotted line represents the proliferation of control lymphocyte cultures without macrophages. Data represent the mean of two independent experiments. (f) *Ex vivo *proliferation (black bar) and viability (white bar) of lymph node cells following a coculture with MBP and myelin. Proliferation was compared to cultures in which no myelin was added. Lymph nodes were isolated 9d-post MBP-immunization. Data represent three independent experiments. Ma: Macrophages, LN: Lymph node, TW: transwell inserts.

To elucidate the mechanisms behind the increased inhibition of lymphocyte proliferation by mye-macrophages, we assessed whether the phagocytosis process as such is responsible for the observed effects on lymphocyte proliferation. For this purpose, macrophages were loaded for 3 consecutive hours with latex beads or zymosan A prior to coculture with lymphocytes. Macrophage treatment with beads or zymosan significantly affected their capacity to modulate lymphocyte proliferation compared to control and myelin treated macrophages (Figure 2a), indicating that the observed increased inhibition of lymphocyte proliferation relies on myelin-specific effects, instead of being induced by the phagocytosis process itself.

Like DCs, macrophages can act as messengers of innate and adaptive immunity by presenting antigen in context of MHC molecules. Mye-macrophages can therefore be assumed to process endogenous myelin and present it to myelin-reactive lymphocytes, possibly influencing reactivity of these lymphocytes. To determine whether the observed immune suppressive effects are dependent on antigen presentation, untreated macrophages and mye-macrophages were cocultured with MBP-reactive lymphocytes in the absence of MBP and irradiated thymocytes. Mye-macrophages did not affect lymphocyte proliferation (Figure 2b), suggesting that, at a macrophage/lymphocyte ratio of 1/10, mye-macrophages do not influence the proliferation of MBP-reactive lymphocytes. These results are in line with the observed equal inhibition of proliferation of both MBP and OVA-reactive lymphocyte by mye-macrophages.

Lipid components of myelin, like cholesterol and arachidonic acid-containing phosphatidylcholine, have been reported to directly inhibit proliferation of ConA stimulated lymphocytes [41,42]. Accordingly, we assessed whether isolated myelin has a direct effect on lymphocyte proliferation. By culturing lymphocytes with increasing concentrations of isolated myelin, in the absence of macrophages, it was established that myelin itself, even at high concentrations, had no significant influence on lymphocyte reactivity (data not shown).

Together, these data indicate that direct effects of myelin on lymphocyte proliferation, myelin-antigen presentation and the phagocytosis process itself are not responsible for the observed effects on lymphocyte proliferation.

### Macrophages inhibit lymphocyte proliferation by the production of nitric oxide

Macrophages have been reported to inhibit lymphocyte proliferation *in vitro *by mechanisms involving inducible nitric oxide synthase (iNOS), arginase I or IDO [43-55]. By administrating either an inhibitor of NOS (L-NMMA), arginase (NOR-NOHA) or IDO (1-MT) to the culture, the involvement of these enzymes was evaluated. Here we demonstrated that an increased activity of both IDO and arginase did not account for the increased inhibition of lymphocyte proliferation by mye-macrophages (Figure 2c). In contrast, administration of L-NMMA completely abrogated the inhibition of lymphocyte proliferation by both macrophages and mye-macrophages. Yet again, cocultures of both OVA and MBP-reactive lymphocytes were affected equally (data not shown). The importance of NO was further delineated by assessment of NO levels in the coculture supernatant of MBP-reactive lymphocytes. In contrast to cocultures of lymphocytes with latex beads or zymosan treated macrophages, a significantly increased concentration of NO was observed in the supernatants derived from cocultures with mye-macrophages, when compared to untreated macrophages (Figure 2d). This increased NO production by macrophages following myelin internalization was absent in monocultures (data not shown), indicating that the observed increased concentration of NO in the coculture with mye-macrophages was induced by lymphocytes. Lymphocyte-derived IFNγ has previously been described to induce NO production in macrophages [56]. However, in our coculture model no increase in IFNγ in the supernatants of cocultures with mye-macrophages compared to untreated macrophages was found (data not shown). Correspondingly, IFNγ neutralization did neither abrogate the increased inhibition of proliferation by mye-macrophages or decrease the NO production in cocultures (data not shown). Nonetheless, when transwell inserts were used to restrict direct cell-cell contact, lymphocyte proliferation in cocultures of both untreated or myelin-treated approached control values (Figure 2e), indicating a role for direct cell-cell contact in the induction of NO.

Noteworthy, when lymph node cells, isolated 9 days post-immunization, were exposed directly to MBP and myelin, an even more pronounced myelin-mediated inhibition of lymphocyte proliferation was observed (Figure 2f). The latter indicates that local lymph node phagocytes show a similar immune suppressive response as peritoneal macrophages following myelin ingestion. Differences detected in proliferation were not due to an altered viability of lymphocytes (Figure 2f).

### Myelin modulates lymph node proliferation *in vivo*

**Figure 3:**
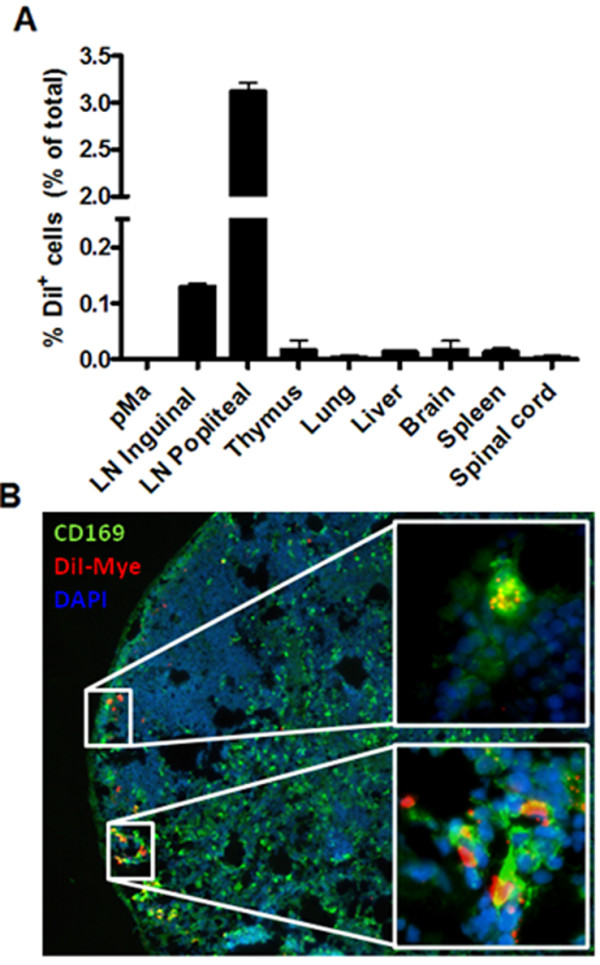
**Myelin-mediated inhibition of lymph node proliferation and myelin migration to CD169^+ ^macrophages in popliteal lymph nodes**. (a) DiI-labeled myelin was injected subcutaneously in the footpad of healthy animals. DiI-flourescene was determined 4d post-injection by flow cytometry. One experiment is shown. (b) Immunohistochemical staining of popliteal lymph nodes 4d post-injection of DiI-labeled myelin. Sections were additionally stained with CD169 and DAPI. pMa: Peritoneal macrophages, LN: Lymph node.

Since we established that myelin internalization by macrophages alters their capacity to modulate T cell proliferation *in vitro*, we assessed the *in vivo *suppressive capacity of mye-macrophages. First, to determine whether subcutaneous injected myelin reaches the draining lymph node and is taken up by macrophages, we injected DiI-labeled myelin in the footpad of healthy animals. A notable migration of myelin towards popliteal lymph nodes was observed (Figure 3a). Immunohistochemical analysis further revealed that myelin was contained primarily in CD169^+ ^macrophages located at the border of the medulla and in the subcapsular sinus (Figure 3b).

**Figure 4:**
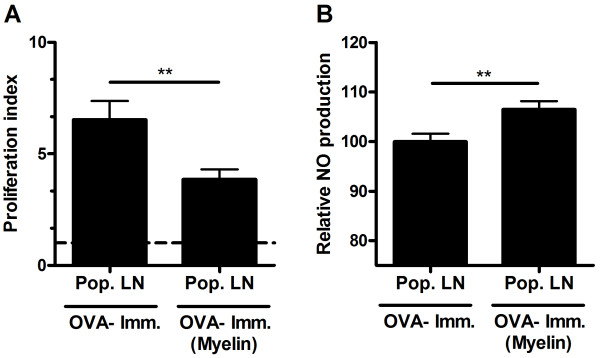
**Myelin inhibits lymphocyte proliferation in OVA-immunized animals**. (a) OVA-immunized animals (N = 7) were treated with myelin at day -4, 0, 4 and 8 (or left untreated). Nine days post-immunization, OVA-reactivity of isolated lymph node cultures was assessed. Non-stimulated cultures were used as control. Data represent the mean of seven independent experiments. (b) NO production by LPS-stimulated popliteal lymph node cultures 9d post-immunization. Lymph node cultures were stimulated with LPS for 18 hr after which NO production in the supernatant was determined. Data represent the mean of seven independent experiments. Pop. LN: Popliteal lymph node.

Next, OVA-immunized animals were treated subcutaneously in the footpad with myelin (d-4, 0, 4, 8 pre/post-immunization). Recall stimulation *in vitro*, 9d post-immunization, revealed a reduced cognate antigen specific proliferation in animals treated with myelin (Figure 4a). Interestingly, LPS-stimulated lymph node cultures from myelin-treated animals demonstrated an increased NO production (Figure 4b). These results demonstrate that myelin is capable of suppressing antigen-specific proliferation *in vivo *and suggest that an increased NO production by these cells is responsible for this effect.

**Figure 5:**
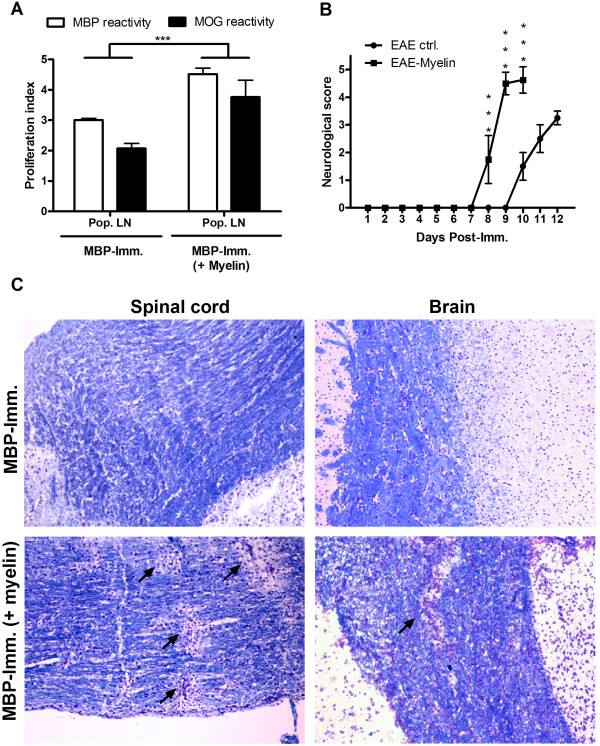
**MBP-immunized animals demonstrate an aggravated disease course following myelin treatment**. (a) MBP-immunized animals were treated with isolated myelin at day -4, 0, 4 and 8 (or left untreated). Ten days post-immunization popliteal lymph node cultures were isolated and MBP (white bars) and MOG (black bars) reactivity was assessed. Non-stimulated cultures were used as control. Data represent the mean of two independent experiments. (b) Neurological score of control (N = 5) and myelin-treated (N = 5), immunized animals was assessed daily. Due to severity of paralysis, myelin-treated animals were sacrificed at d10. (c) LFB/cresyl violet staining of spinal cord (left) and brain tissue (right) of myelin-treated animals at day 10. Arrows depict demyelinated regions. One experiment is shown. Pop. LN: Popliteal lymph node.

In contrast to OVA-immunized animals, MBP-immunized animals revealed an increased reactivity towards MBP and MOG following myelin treatment (Figure 5a). Moreover, myelin-treated animals demonstrated an earlier onset of paralysis and a more severe neurological score at the peak of disease (Figure 5b). In concordance, a LFB/cresyl violet staining of brain and spinal cord sections demonstrated increased cellular infiltrates and the presence of demyelinated areas in myelin-treated animals (Figure 5c). Noteworthy, when MBP-immunized animals were treated with latex beads no overt effect on disease score and onset was found (data not shown), indicating that the aggravated disease score following myelin treatment is myelin-specific.

**Figure 6:**
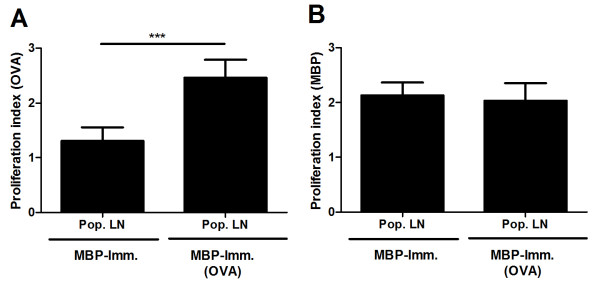
**OVA does not influence lymphocyte reactivity to MBP in MBP-immunized animals**. (a, b) MBP-immunized animals (N = 4) were treated with OVA at day -4, 0, 4 and 8 (or left untreated). Nine days post-immunization, OVA- (a) and MBP (b) reactivity of isolated lymph node cultures was assessed. Non-stimulated cultures were used as control. Data represent the mean of four independent experiments. Pop. LN: Popliteal lymph node.

To exclude the possibility that in the restricted area of the lymph node the availability of OVA in OVA-immunized animals is altered by the additional presence of myelin proteins, hereby reducing OVA-specific proliferation, MBP-immunized animals were treated subcutaneously with OVA. As expected, an increased reactivity towards OVA was observed in isolated lymph node cells (Figure 6a). However, MBP-specific proliferation was unchanged (Figure 6b), demonstrating that the observed myelin-mediated inhibition of proliferation of lymph nodes cultures from OVA-immunized is independent of a reduced availability of OVA by myelin proteins.

These results indicate that mye-macrophages have a dual influence on proliferation, depending on the nature of surrounding lymphocytes. On one hand they aggravate autoimmunity by activating myelin-reactive lymphocytes and on the other hand they have the capacity to suppress lymphocyte activation by the secretion of NO.

## Discussion

In this study we have established that macrophages that have phagocytosed myelin modulate the proliferation of autoreactive T cells. The observed inhibition of TCR-triggered lymphocyte proliferation by mye-macrophages was antigen-independent, as both OVA- and MBP-reactive lymphocytes show an identical reduction in proliferation following coculture with mye-macrophages *in vitro*. Additionally, when *in vivo *primed lymph node cultures were restimulated directly *in vitro *in the presence of myelin, an even more pronounced immune suppression was observed. These results indicate that both macrophages and lymph node phagocytes obtain immune suppressive properties following myelin internalization.

Macrophages may inhibit proliferation of lymphocytes in various manners, including IDO-mediated depletion of tryptophan, arginase-mediated lowering of L-arginine and lymphocyte CD3ζ expression, and NO-mediated reduction of tyrosine residue phosphorylation in the Jak3/STAT5 pathway and inhibition of caspase activity [43-52]. We demonstrate that the non-selective iNOS inhibitor L-NMMA completely reversed the observed inhibition of proliferation by both control and mye-macrophages while the other pathways were not involved. In line with this, an increased concentration of NO was demonstrated in the coculture supernatant of mye-macrophages, explaining the observed inhibition of lymphocyte proliferation by mye-macrophages.

Abrogation of direct cell-cell contact restored lymphocyte proliferation in our cocultures. This finding, together with the observed role of NO in the inhibition of lymphocyte proliferation, suggests that direct contact between both cell types is a necessary prerequisite for stimulating NO-mediated inhibition of lymphocyte proliferation by macrophages. On the other hand, NO might, due to extreme short half-life, not reach lymphocytes when direct contact is restricted. Future studies should therefore determine the mechanism behind the macrophage- and mye-macrophage-mediated inhibition of lymphocyte proliferation in our cocultures. Although lymphocyte-derived IFNγ is described to induce NO production by macrophages, we were unable to demonstrate a role for lymphocyte-produced IFNγ in the observed inhibition of lymphocyte proliferation [56].

As we demonstrated an increased, NO-mediated inhibition of lymphocyte proliferation by mye-macrophages *in vitro*, myelin-rich phagocytes in secondary lymph nodes might fulfill an identical suppressive role *in vivo*. CD169^+ ^macrophages in lymph nodes are described to be primarily involved in uptake and relay of viral particles and immune complexes, and activation of follicular B lymphocytes [57-60]. We demonstrate that a subcutaneous injection of myelin in the footpad results in a notable migration of myelin towards CD169^+ ^medullary and subcapsular sinus (SCS) macrophages in popliteal lymph nodes. Given the abundance of lipids in myelin, these results are in line with a recent report showing active phagocytosis of lipid-coated silica particles by SCS macrophages [61].

To explore the possible immune suppressive properties of mye-macrophages *in vivo*, OVA-immunized animals were treated subcutaneously in the footpad with myelin. Restimulated popliteal lymph nodes of myelin-treated animals display reduced OVA-induced proliferation compared to lymphocytes derived from untreated OVA-immunized animals. This effect is independent of interference of myelin proteins on OVA antigen presentation, as lymph node cultures of MBP-immunized animals treated subcutaneously with OVA did not reduce MBP reactivity. These results demonstrate that mye-macrophages suppress lymphocyte proliferation *in vivo*. In contrast, lymph node cultures derived from MBP-immunized animals that were treated with myelin showed an enhanced proliferative capacity. Although we demonstrated that mye-macrophages are unable to increase proliferation of MBP-reactive lymphocytes *in vitro*, the presence of other myelin-rich antigen-presenting cells, like migrated langerhans cells and local lymph node DCs, might explain the increased reactivity against MBP and MOG. Furthermore, B cells have been described to capture antigen-containing immune complexes from SCS macrophages processes and migrate to the T cell zone to influence antigen presentation [57,60]. Finally, the discrepancy in literature regarding the skewing of macrophages following myelin internalization suggests that myelin can have divergent effects on macrophage polarization and its APC-like or immune suppressive properties, which may depend on the macrophage origin and local environmental stimuli. Likewise, the nature of surrounding lymphocytes, for example being myelin-protein, non-myelin or myelin-lipid specific, might determine whether the presence of mye-macrophages results in stimulation or suppression of lymphocyte activity. Future studies should therefore determine whether lymphocytes surrounding mye-macrophages in CNS draining lymph nodes recognize antigen presented by these cells and are hereby activated.

Interestingly, we demonstrated an increased capacity of lymph nodes cells from myelin treated, OVA-immunized animals to produce NO following LPS stimulation. These results indicate a direct role of macrophage-produced NO in the observed decrease in OVA reactivity in myelin-treated animals, as observed *in vitro*. The importance of NO in the control of inflammation in EAE is supported by studies showing an aggravation or inability to recover following treatment with an iNOS inhibitor in respectively the induction or the remission phase of EAE [62,63]. Likewise, treatment with the NO-donor SIN-1 during the induction phase of EAE ameliorated EAE, which was correlated with a reduced immune cell infiltration and antigen-induced proliferation [64]. Finally, EAE insusceptibility in rat strains like the Piebald Virol Glaxo and the Brown Norway strain was correlated with an increased production of immune suppressive NO following immunization [65,66]. These results demonstrate that NO displays a disease-mitigating role in EAE by inhibiting lymphocyte proliferation. Based on these and our findings, we suggest that mye-macrophages in the perivascular space and CNS-draining lymph nodes can fulfill a suppressive role in MS by producing NO, hereby silencing autoreactive lymphocytes.

It is unclear which myelin components are responsible for the observed immune suppressive effects. To date, despite the abundance of lipids in myelin, most studies have mainly focused on the role of myelin proteins in neurodegenerative diseases. Interestingly, several lipids present in myelin have been reported to alter macrophage signaling and transcription. Intracellular, lipid sensors like LXR and PPAR, which are respectively activated by cholesterol derivates and non-esterified fatty acids, have recently been described as key regulators of lipid metabolism and inflammation, and may be activated following myelin internalization [67-69]. Similarly, individual lipids present in myelin can alter the macrophage or microglial response by binding to specific receptors and activating or blocking signalling cascades pivotal in inflammation [70-73].

Macrophages can adopt divergent phenotypes based on specific stimuli in their microenvironment [74-77]. Moreover, mye-macrophages have been described to display divergent phenotypes depending on the location in the lesion, suggesting that they are likely to exert diverse functions depending on their micro-location [12]. By using thioglycolate-elicited PECs, as a representative model for infiltrating monocytes in EAE and MS, we established that myelin internalization results in an altered macrophage function, characterized by an increased production of NO [78]. These mye-macrophages may have dual effects during MS pathogenesis. Whereas NO production by mye-macrophages can negatively influence neuronal integrity and block axonal conduction locally in the brain parenchyma, we show that NO also suppresses lymphocyte proliferation. Thus, depending on the surrounding cells, mye-macrophages can be involved in either limiting or promoting autoimmune-mediated demyelination.

## Conclusion

We demonstrate that myelin phagocytosis leads to an altered macrophage function that inhibits lymphocyte proliferation. The observed immune suppression was mediated by an increased production of NO by mye-macrophages. Additionally, we establish that mye-macrophages fulfill an ambiguous role *in vivo*. On one hand mye-macrophages aggravate autoimmunity by activating myelin-reactive lymphocytes in secondary lymph nodes and on the other hand they suppress lymphocyte reactivity by producing NO.

## Competing interests

The authors declare that they have no competing interests.

## Authors' contributions

BJ performed the experiments, analyzed the data and wrote the manuscript. HJ, HN and SP participated in its design and coordination, and have been involved in revising the manuscript. All authors have read and approved the final version of this manuscript
